# Ionomic and transcriptomic analyses of two cotton cultivars (*Gossypium hirsutum* L.) provide insights into the ion balance mechanism of cotton under salt stress

**DOI:** 10.1371/journal.pone.0226776

**Published:** 2019-12-23

**Authors:** Huijuan Guo, Shuangnan Li, Wei Min, Jun Ye, Zhenan Hou

**Affiliations:** Department of Resources and Environmental Science, Shihezi University, Shihezi, Xinjiang, People’s Republic of China; USDA-ARS Southern Regional Research Center, UNITED STATES

## Abstract

Soil salinity is a major abiotic stress factor that limits cotton production worldwide. To improve salt tolerance in cotton, an in-depth understanding of ionic balance is needed. In this study, a pot experiment using three levels of soil salinity (0%, 0.2%, and 0.4%, represented as CK, SL, and SH, respectively) and two cotton genotypes (salt-tolerant genotype: L24; salt-sensitive genotype: X45) was employed to investigate how sodium chloride (NaCl) stress effects cotton growth, ion distribution, and transport, as well as to explore the related mechanism. The results showed that SL treatment mainly inhibited shoot growth, while SH treatment caused more extensive impairment to roots and shoots. The growth inhibition ratio of NaCl stress on X45 was more marked than that of L24. Under NaCl stress, the Na concentration in the roots, stems and leaves significantly increased, whereas the K, Cu, B, and Mo concentration in roots, as well as Mg and S concentrations in the leaves, significantly decreased. Under salt stress conditions, salt-tolerant cotton plants can store Na in the leaves, and as a result, a larger amount of minerals (e.g., Cu, Mo, Si, P, and B) tend to transport to the leaves. By contrast, salt-sensitive varieties tend to accumulate certain minerals (e.g., Ca, P, Mg, S, Mn, Fe, Cu, B, Mo, and Si) in the roots. Most genes related to ion transport and homeostasis were upregulated in L24, but not in X45. The expression level of *GhSOS1* in X45 was higher than L24, but *GhNHX1* in L24 was higher than X45. Our findings suggest that the two varieties response to salt stress differently; for X45 (salt-sensitive), the response is predominantly governed by Na^+^ efflux, whereas for L24 (salt-tolerant), vacuolar sequestration of Na^+^ is the major mechanism. The expression changes of the genes encoding the ion transporters may partially explain the genotypic difference in leaf ion accumulation under salt stress conditions.

## Introduction

Soil salinity is a serious threat to agricultural productivity worldwide, and is an important factor that reduces crop production. Recent estimates have indicated that approximately 1128 million ha of global land is affected by salinity and sodicity [1, 2]. Severe salt stress destroys plant cell membrane structure, inhibits photosynthesis, produces toxic metabolites, and reduces nutrient absorption, leading to obstruction of plant growth, decline in productivity, and even death [3, 4]. Cotton is an important industrial crop that is widely used for fiber and oil industries around the world. As a moderately salt-tolerant crop, cotton is considered as a "pioneer crop" for the development and utilization of salt soil and a model crop to understand salinity tolerance [5].

Soil salinity stress inhibits plant growth and development by means of osmotic stress, ion toxicity, and the resulting nutrient imbalance [6]. Salt stress can destroy the balance between water potential and the ion distribution between crops and soils, thus leading to nutrient deficiency and cell metabolism disorders [4, 7–9]. Salt stress inhibits the uptake of large amounts of elements (e.g., N, P, K, Ca, Mg, and S) by crops, as well as limits the absorption of some trace elements (e.g., Fe, Cu, Zn, Mn, and B) [10]. In a high salt concentration environment, crops respond to nutritional imbalance by regulating ion transport and maintaining ionic homeostasis. These mineral elements act as nutrients for the growth of crops, and also participate in various physiological metabolic processes that directly or indirectly affect the salt tolerance of crops in a variety of ways. Salt stress harm is due to excessive amounts of Na^+^ in the soil, which causes sodium ion toxicity and breaks the original ion balance in plants. Therefore, maintaining stable intracellular mineral ionic content and establishing a new ion homeostasis represent the major mechanisms for cotton to adapt to salt stress [11].

Cotton plants respond to salt stress by maintaining the balance of K^+^ and Na^+^ ions in their tissues; maintaining a higher K^+^/Na^+^ ratio in tissues is considered more important than simply maintaining a lower Na^+^ concentration [12]. In addition, Ca and Mg are also thought to be important for improving salt tolerance in cotton [13]; however, Severino *et al*. [14] showed that Ca and Mg could not reduce Na^+^ toxicity in cotton at the seedling stage. Although many studies investigating the response of cotton to salt stress and the improvement of salt tolerance by mineral elements have been conducted [15, 16], most of these studies have focused on the effects of salt stress on one or several mineral elements, and the responses to other elements and their dynamic changes to salt stress have not been fully elucidated [9]. The ionome is defined as the mineral nutrients and trace element composition of an organism and represents the inorganic components of cellular and biological systems [10, 17]. Ionomics is the study of ionomes using high-throughput analytical methods (ICP-AES, ICP-MS, and XRF) to quantify the elemental characteristics of organisms or tissues. Ionomics represents an important research approach for understanding element-element, element-gene, and element-environment relationships, as well as the physiological and biochemical functions of one certain element [18]. It is important to note that ionomes are under tight genetic control and vary among tissues [19].

Environmental factors can influence ion homeostasis through regulating the related genes. In cotton, the *GhAKT1* and *GhKT2* genes regulate Na^+^ absorption and transport to the aboveground plant parts, thereby maintaining K^+^ balance [20]. The Salt Overly Sensitive (SOS) pathway is involved in the maintenance of ionic homeostasis, in which ion transporters such as *SOS1* are regulated by the calcium-responsive protein kinase *SOS3-SOS2* complex [21]. *SOS3* is a myristoylated calcium-binding protein and senses changes in cytosolic calcium during salt stress [22, 23]. *GhNHX1* participates in Na^+^ transport and compartmentalization, maintains osmotic balance, and reduces cytosolic Na^+^ concentration [24]. *GhVP* and H^+^-ATP participate in the transport of multiple cations and provide energy [25]. Although the abovementioned K^+^/Na^+^ ion balance-related genes play important roles in the steady state plasmon remodeling of cotton under salt stress, there might be many unidentified but equally important genes related to the absorption and transport of other elements. RNA-seq analysis has been extensively utilized to determine global expression patterns under abiotic stress, thereby allowing a comprehensive identification of differentially expressed genes [26, 27]. Under salt stress, many genes, which either directly protect the plant from salt stress or regulate the expression of other target genes, are induced. Therefore, the transcriptome is typically used together with the ionome to reveal the molecular mechanism of ion balance.

An in-depth understanding of ionic homeostasis is imperative for the improvement of salt tolerance in cotton. However, the ionic homeostasis of elements and their relationship under NaCl stress have not been fully elucidated in cotton. In this study, to better understand the ion balance mechanism of cotton to salt stress, we used two varieties of cotton that differ in salt tolerance (salt-tolerant, L24; salt-sensitive, X45) and investigated changes in the ionome (roots, stems, and leaves) and transcriptome (leaves) in response to NaCl stress at the seedling stage. Moreover, the correlations between Na and other ionic contents in tissues of salt-tolerant and salt-sensitive cotton were analyzed under NaCl stress. The main objectives of this pot experiment were to: (i) investigate changes in the cotton ionome in response to NaCl stress; (ii) determine differences in tissue ionome responses to salt stress between genotypes; and (iii) provide insights into the Na^+^ and K^+^ transport-related genes of ionic homeostasis and transcriptome responses to salt stress in cotton.

## Materials and methods

### Experiment site and design

The pot experiment was conducted in the greenhouse of the agricultural experimental station of Shihezi University in 2017. The experimental soil was collected from the experimental station farm, and the soil depth was 0–30 cm. The soil at the site was alluvial, gray desert soil, classified as a calcaric fluvisol by FAO/UNESCO. Some of the soil physical and chemical properties were as follows: electrical conductivity (EC) in 1:5 (soil:water) extract, 0.17 dS·m^−1^; pH: 8.16; organic matter: 6.77 g·kg^−1^; total nitrogen: 0.57 g·kg^−1^; available phosphorus: 7.21 mg·kg^−1^; and available potassium: 182 mg·kg^−1^. The two cotton genotypes used were Lu-mian-yan No. 24 (a salt-tolerant cotton genotype, L24) and Xin-lu-zao No. 45 (a salt-sensitive cotton genotype, X45).

The experiment consisted of three soil salinity levels [0.17, 0.76 and 1.39 dS·m^−1^ (EC_1:5_, electrical conductivity of 1:5 soil/water extract), represented as CK, SL, and SH, respectively, thereinafter]. The detailed steps were as follows: different concentrations of NaCl were added to naturally dried, crushed, and sieved (2-mm pore size) soil (with salt/dry soil weight ratios of 0%, 0.20%, and 0.40%, respectively) to produce a supersaturated state (the same volume of deionized water was added in control plants) and left to stand for 1 month. Then, the three salinity levels of the naturally dried, crushed, and sieved soil were determined. A metal cylinder with an internal diameter of 20 cm and a height of 60 cm was used. The experimental soil was layered at a 50-cm depth according to the soil bulk density of 1.25 g·cm^−3^, 10 cm for each layer, and 20 kg for each pot. Each treatment had 6 replications. Water was applied by drip irrigation method at a rate of 2.1 L·h^−1^ per emitter. The drip irrigation pipe was laid flat on the pot, with each pot supplied by an emitter, and the emitter was fixed at the center of the top of the pot.

Cotton seeds were planted on May 6, 2017, and each pot was sowed with 20 seeds. Each pot was drip-irrigated with 3 L of water at sowing to improve germination. When the cotton seedlings grew to show "2 leaves and 1 heart", four cotton seedlings with similar growth statues were kept on each pot. To ensure water supply, water replenishment was conducted at regular intervals during the experiment, so that the soil moisture was maintained at 60%–80% of the field capacity.

### Sample collection and treatment

#### Growth analysis

The dry matter of cotton was sampled and measured, and three representative cotton plants were selected for each treatment. The samples (roots, stems, and leaves) were separated from the indoors, and were green killed at 105°C. After 30 min, the samples were oven-dried at 70°C for 72 h, weighed, ground to pass a 1-mm sieve, and stored at room temperature.

#### Ionome analysis

The specific steps of plant ionome analysis were as follows: samples were ground and 0.25 g of each sample was collected. After adding 10 m L of concentrated nitric acid, the samples were digested in a microwave digestion instrument (Milestone, ETHOS A). After microwave digestion, the samples were placed on a 230°C electric heating plate for acid driving for about 20 min. After the digestion tank was removed, the solution was then transferred to a 25-mL colorimetric tube with ultrapure water. The microwave digestion tank and the lid were rinsed 3–5 times, and the buffer solution was transferred to a colorimetric tube, diluted to volume, and then shaken evenly. The ionome concentration (Na, P, K, Ca, Mg, S, Fe, B, Mn, Zn, Cu, Mo, and Si) in the roots, stems, and leaves were measured using inductively coupled plasma mass spectrometry (ICP-MS, ICAP-Q series, Thermo Fisher Scientific, USA).

#### *GhSOS1*, *GhNHX1*, *GhHKT1* and *GhAKT1* gene expression analysis

Gene expression was measured using real-time quantitative PCR. The samples (roots and leaves) were homogenized in liquid nitrogen before isolation of RNA. Total RNA was isolated using TRIZOL REAGENT(Tiangen, Beijing) following the manufacturer’s instructions. cDNA was synthesized using RevertAid^™^ First Strand cDNA Synthesis Kit (Thermo Scientific). Gene-specific primers were designed using Primer software (ver. 5.0), The primers used in qRT-PCR analysis are listed in S1 Table. qRT-PCR was performed on an ABI PRISM 7300 Sequence Detection System using 0.1 μL cDNA, 5 μL SYBR Premix Ex Taq II (Takara, Dalian, China). 0.4 μL of each primer (forward and reverse, concentration: 10 μmol/L), and H_2_O were added to a final reaction volume of 10 μL. The qPCR conditions were as follows: preincubation at 95°C for 5 min, followed by 40 cycles of 95°C for 15 s and 60°C for 1 min. The relative expression levels of each gene were calculated using the 2^-ΔΔCT^ method.

#### Illumina sequencing and real-time quantitative PCR (qRT-PCR) analysis

The leaves of cotton seedlings subjected to NaCl stress were simultaneously collected from each individual plant and were frozen in liquid nitrogen and stored at -80°C. The amplicons were sequenced with the Illumina HiSeq^™^ 2000 system (Biomarker Technology Co., Ltd., Beijing, China). The specific steps are: RNA sample purification, synthesis of double-stranded cDNA, joint addition, amplification of DNA library, library quality detection, homogenization library and some other steps, and then transcriptome sequencing. The raw sequencing data was deserialized to obtain the raw reads; the low-quality reads were removed; and the transcriptome data were reassembled from scratch using the Velvet/Oases software. Gene function was annotated based on the following databases: Nr (NCBI non-redundant protein sequences); Nt (NCBI non-redundant nucleotide sequences); Pfam (Protein family); KOG/COG (Clusters of Orthologous Groups of proteins); Swiss-Prot (a manually annotated and reviewed protein sequence database); KO (KEGG Ortholog database); and GO (Gene Ontology). We used a specified genome as reference for sequence alignment and subsequent analysis. The reference genome was downloaded from http://mascotton.njau.edu.cn.

To verify the reliability of our high-throughput sequencing results, 11 genes were selected for real-time quantitative PCR. The methods were the same as *GhSOS1*, *GhNHX1*, *GhHKT1* and *GhAKT1* gene expression analysis. The primers used in qRT-PCR analysis are listed in S1 Table

#### Data analysis

The relative biomass and growth inhibition rate were calculated based on the following equations [28]:

Growthinhibitionratio(%)=(Dryweightofthecontrolplants-Dryweightofthetreatedplants)/Dryweightoftheblankplants×100%.

The data were analyzed using the SPSS 17.0 software. Means (n = 3) and standard errors (SD) were calculated. One-way ANOVA was used to determine significant differences among treatments. Duncan multiple range tests were conducted to determine whether there were significant differences among individual treatments at *P*<0.05 level.

Statistical differences in the growth and ion concentrations in the cotton tissues were assessed by variance (ANOVA), and the means were compared using Duncan’s multiple range test. A significance level of *P*<0.05 was applied. Pearson’s correlation analyses of mineral concentrations in different tissues were performed using a significance level of *P* < 0.05. Using the CANOCO 4.5 software, PCA was performed to visualize the ionomic profile of the two cotton genotypes and the situations between different plant parts. Differential expression analysis was performed using the R package DEGseq. All statistical calculations were performed using the SPSS 17.0 software.

## Results and analysis

### Biomass and growth inhibition ratio

**Fig 1:**
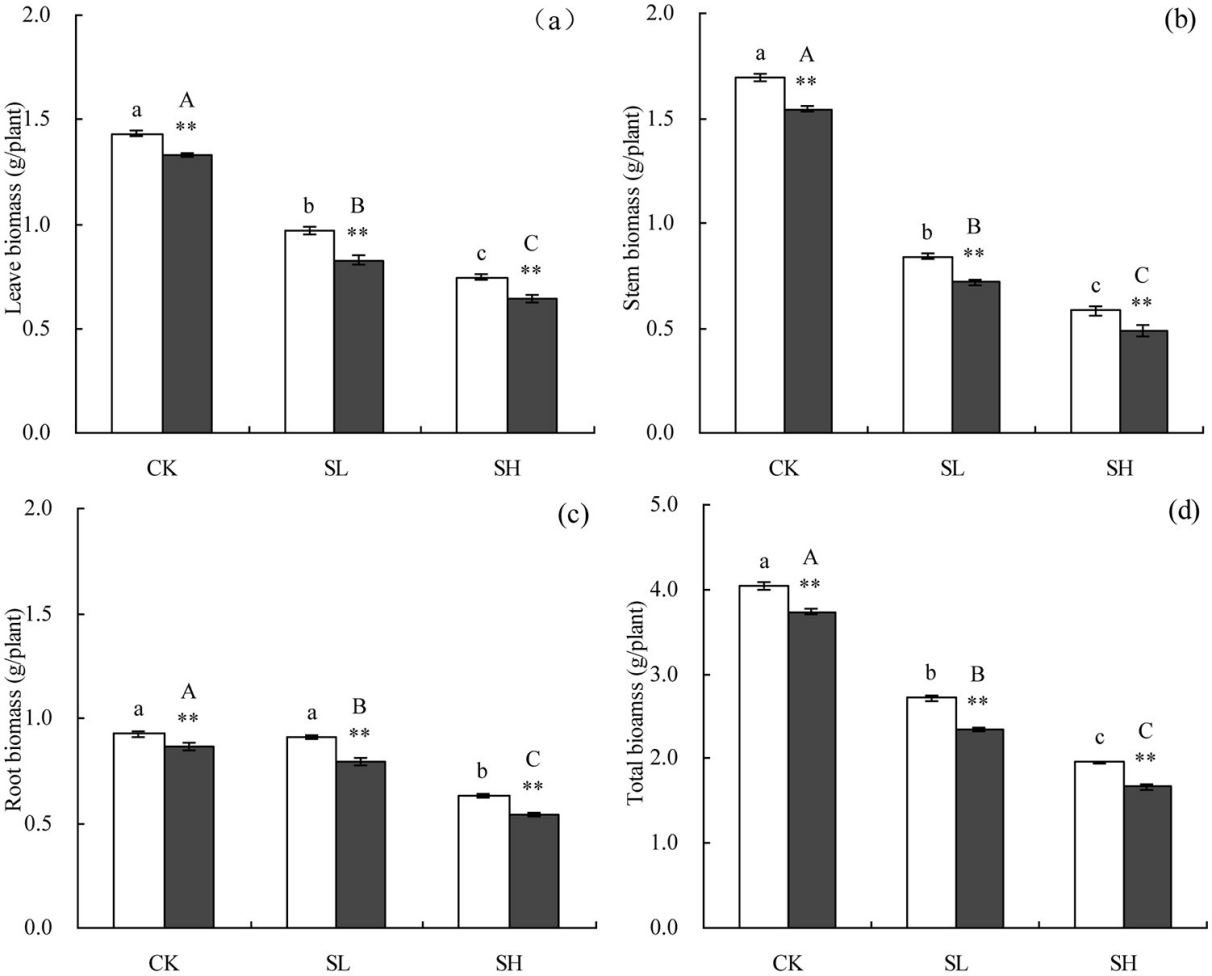
Effect of NaCl stress on the relative biomass of cotton. Symbols indicate L24 cultivar (white bar) and X45 cultivar (black bar). Vertical bars represent ± standard error (n = 3). Asterisks indicate a significant difference between L24 and X45 (*P<0.05; **P<0.01). Bars labeled with the different lowercase letters on open square bars or uppercase letters on closed square bars are significantly different (P<0.05). (a), (b), (c), and (d) indicate biomass in the roots, stems, leaves, and total plant, respectively.

The effects of NaCl stress on cotton biomass are presented in Fig 1. In general, cotton biomass decreased as soil salinity increased. Compared with the CK treatment, SL treatment reduced total cotton biomass by 35.02%. Similarly, SH treatment reduced total cotton biomass by 53.52%. In most cases, the biomass of L24 tissues was significantly higher than those of X45. The average of two soil salinities, the biomass of the leaves, stems, roots, and total of L24 were significantly higher than those of X45 by 16.89%, 18.08%, 15.46%, and 16.78%, respectively.

**Fig 2:**
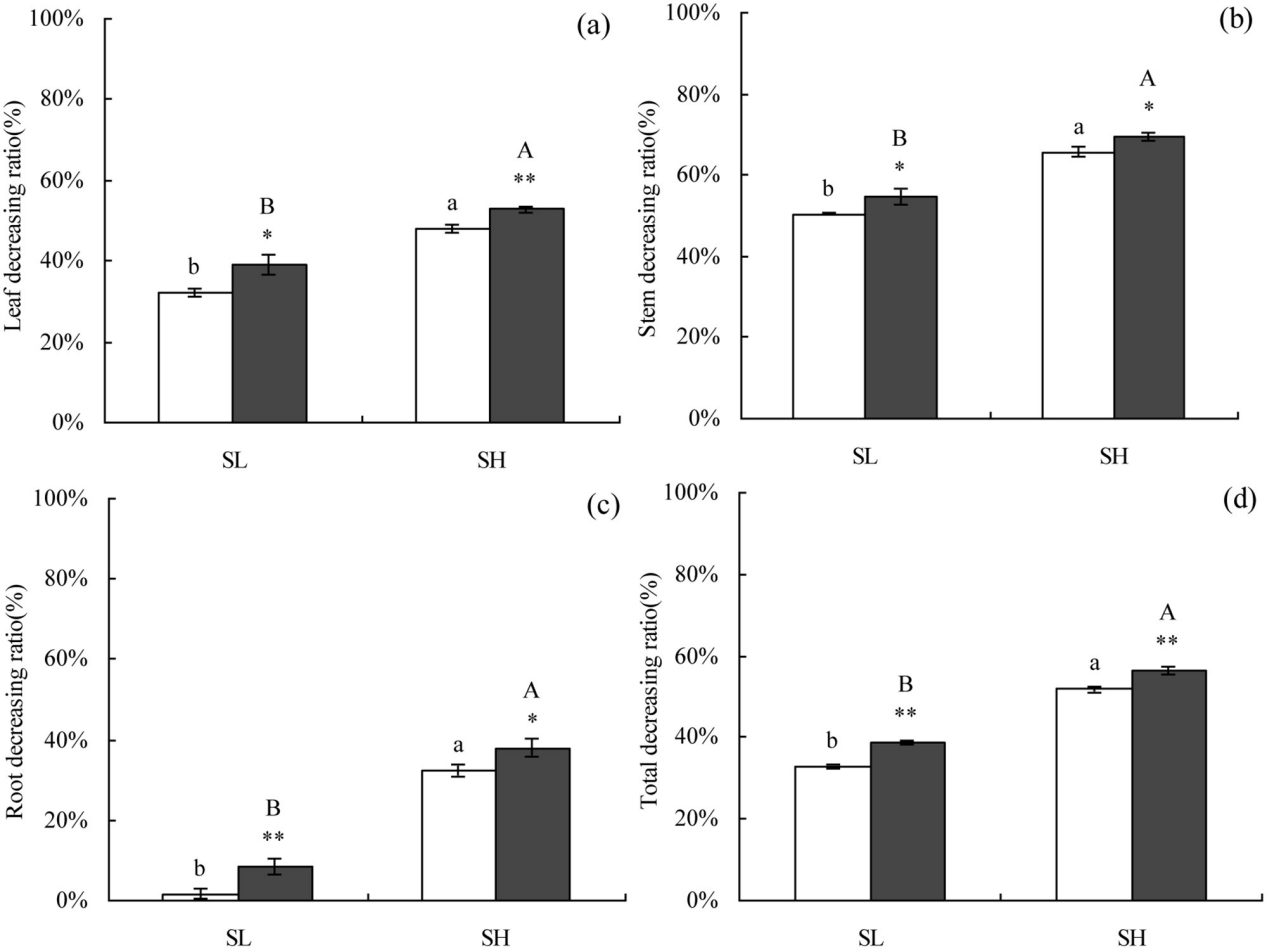
Effect of NaCl stress on growth decreasing ratio of cotton. Symbols indicate L24 cultivar (white bar) and X45 cultivar (black bar). Vertical bars represent ± standard error (n = 3). Asterisks indicate a significant difference between L24 and X45 (*P<0.05; **P<0.01). Bars labeled with the different lowercase letters on open square bars or uppercase letters on closed square bars are significantly different (*P*<0.05). (a), (b), (c), and (d) indicate the growth inhibition rate in the roots, stems, leaves, and total, respectively.

Not surprisingly, NaCl stress significantly inhibited cotton growth, and the growth inhibition ratio of the roots, stems, and leaves of the two cultivars were significantly increased with greater NaCl stress. In general, growth inhibition under NaCl stress of X45 was higher than that of L24 (Fig 2). The average of two soil salinities, the growth inhibition rates of the leaves, stems, and roots, and total of L24 was significantly lower than those of X45 by 12.49%, 6.54%, 26.58%, and 10.83%, respectively. Regardless of cotton genotype, under SL treatment, the growth inhibition rate of the leaves, stems, and roots, and total was 35.70%, 52.54%, 4.99%, and 35.68%, respectively. Under SH treatment, the growth inhibition rate of the leaves, stems, and roots, and total was 50.42%, 67.49%, 35.05%, and 54.00%, respectively. The above results indicate that low-concentration NaCl stress mainly inhibited the growth of shoots (stems and leaves), while high-concentration NaCl stress significantly inhibited shoot and root growth.

### Ionomic responses to NaCl stress in different cotton tissues

**Fig 3:**
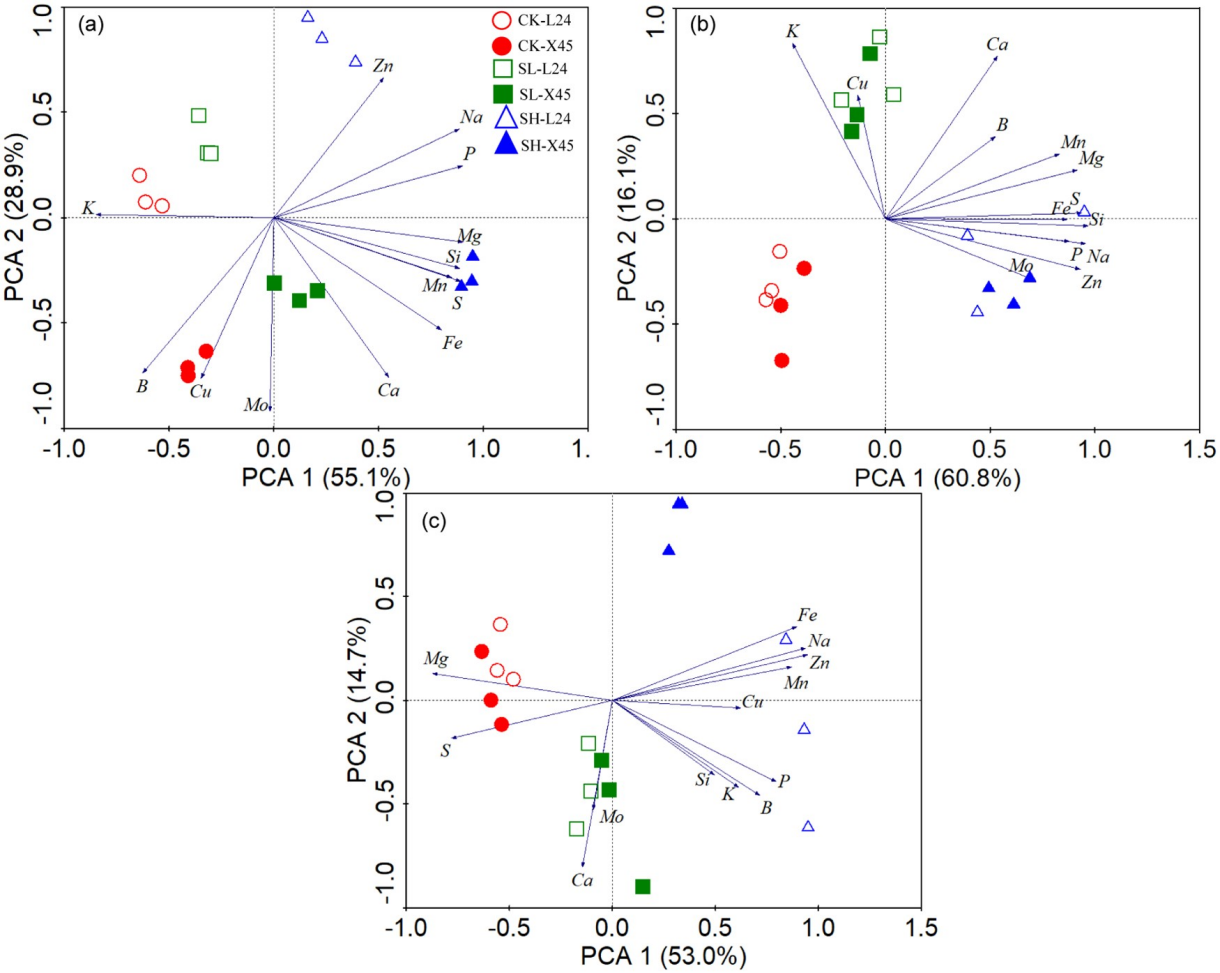
Tissue ionome variation analysis using PCA at the seedling stage and the loadings of elements to the PC1 and PC2. (A) Root ionome variations among samples and the loadings of elements to the PC1 and PC2 in roots; (B) stem ionome variation among samples and the loadings of elements to the PC1 and PC2 in shoots. (C) Leaf ionome variation among samples and the loadings of elements to the PC1 and PC2 in shoots.

To demonstrate the effect of NaCl stress on element distribution in cotton seedlings, we analyzed the concentration of Na, K, P, Ca, Mg, S, Zn, Mn, Fe, Cu, B, Mo, and Si in the roots, stems, and leaves under NaCl stress (Table 1). The PCA results of cotton roots, stems, and leaves under NaCl stress are shown in Fig 3, and it is clear that our analysis effectively distinguished different NaCl concentration gradients and cotton genotypes. Different NaCl stress gradients were well separated in the first principal component, accounting for 55.1%, 60.8%, and 53.0% of the total coefficient of variation in the roots, stems, and leaves, respectively. The major elements that contributed to PC1 were Na, Mg, S, P, and Si in the root ionome; Na, Si, Zn, S, and Mg in the stem ionome; and Na, Mg, Zn, Fe, and Mn in the leaf ionome. Root ionome analysis demonstrated that L24 and X45 could be clearly distinguished using the second principal component, which explained 28.9% of the total coefficient of variation, while stem ionome analysis could not, indicating that there was no significant difference in terms of the stems between the two varieties. Leaf ionome analysis showed that L24 and X45 were well distinguished under SH treatment, which explained 14.7% of the total coefficient of variation. The contribution of elements to the PC2 was predominated by Ca, Cu, Zn, and Mo in the root ionome; K, Ca, and Cu in the stem ionome; and Ca, Mo in the leaf ionome.

**Table 1:** Effects of NaCl stress on the concentration of mineral elements (mg g^-1^ DW) in roots, stems and leaves of two cotton varieties.

Tissue	Genotype	Treatment	Na	K	P	Ca	Mg	S	Zn	Mn	Fe	Cu	B	Mo	Si
Root	L24	CK	0.666c	12.20a	0.957e	3.158b	2.126c	2.674c	0.028d	0.016d	0.639d	0.013bc	0.021b	0.007d	0.194c
		SL	1.804b	12.19a	1.222c	3.098b	2.251c	2.644c	0.034cd	0.017d	0.656d	0.010d	0.017d	0.008c	0.207c
		SH	3.343a	9.969b	1.389b	3.065b	2.637b	2.852c	0.210a	0.022bc	0.858c	0.011d	0.014e	0.004e	0.205c
	X45	CK	0.755c	11.77a	1.057de	3.633a	2.261c	2.711c	0.029d	0.020c	0.987b	0.017a	0.024a	0.012a	0.195c
		SL	1.776b	9.827b	1.136cd	3.594a	2.392bc	3.267b	0.042c	0.024ab	0.985b	0.014b	0.019c	0.010b	0.229b
		SH	3.441a	9.379b	1.594a	3.897a	3.942a	3.841a	0.091b	0.027a	1.212a	0.012cd	0.016d	0.009b	0.280a
Stem	L24	CK	0.417c	13.02c	1.114c	4.857d	2.965d	2.979c	0.015b	0.004c	0.040c	0.005bc	0.010a	0.003bc	0.035c
		SL	1.802b	14.58b	1.181bc	6.480a	3.870bc	3.467bc	0.034b	0.006b	0.129b	0.006a	0.011a	0.004bc	0.051b
		SH	5.692a	11.55d	1.472a	5.785c	4.457a	3.950ab	0.156a	0.006ab	0.248a	0.005b	0.011a	0.005ab	0.071a
	X45	CK	0.355c	11.75d	1.085c	4.497e	3.087d	3.297c	0.015b	0.004c	0.055c	0.005bc	0.010a	0.003c	0.044bc
		SL	1.998b	15.49a	1.169bc	6.223b	3.759c	3.466bc	0.028b	0.006ab	0.062c	0.004bc	0.012a	0.003c	0.050b
		SH	5.608a	10.42e	1.3450ab	5.932c	4.255ab	4.032a	0.136a	0.008a	0.153b	0.003c	0.012a	0.007a	0.072a
Leaves	L24	CK	0.917de	14.67b	1.399b	26.669bc	6.705a	18.143b	0.028c	0.036c	0.348c	0.005b	0.039b	0.013ab	0.165c
		SL	1.983c	19.21a	1.507b	28.748a	6.148b	15.693c	0.039b	0.043b	0.341c	0.004c	0.043b	0.013ab	0.183bc
		SH	8.838a	19.15a	1.744a	26.022c	5.211c	14.538c	0.069a	0.055a	0.510a	0.005a	0.055a	0.014a	0.215a
	X45	CK	0.651e	14.98b	1.402b	25.906c	6.887a	20.957a	0.027c	0.042b	0.330c	0.003d	0.043b	0.013ab	0.197ab
		SL	1.634cd	21.08a	1.649a	28.366ab	6.327b	18.022b	0.040b	0.045b	0.384b	0.004c	0.052a	0.011bc	0.182bc
		SH	6.168b	19.07a	1.476b	25.396c	6.336b	14.837c	0.063a	0.053a	0.495a	0.004c	0.043b	0.009c	0.177bc

Different letters in the same column for the roots, stems, or leaves indicate significant differences (*P*<0.05) between individual treatments.

In response to NaCl stress, compared with the CK treatment, the averaged Na concentration of the two cultivars in the roots, stems, and leaves increased by 2.52-, 4.92-, and 2.30-fold under SL treatment, and 4.77-, 14.64-, and 9.57-fold under SH treatment (Table 1). In addition, the averaged concentration of K, Cu, B, and Mo in the roots significantly decreased by 5.55%, 20%, 20%, and 5.2% with SL treatment, and by 17.0%, 23.33%, 33.33%, 31.58% with SH treatment, respectively. The concentration of Mg and S in the leaves significantly decreased by 8.22% and 13.77% with SL treatment, and 15.0% and 24.87% with SH treatment, respectively. Generally, the contents of K, Cu, and B decreased in the roots and increased in the leaves, indicating that K, Cu, and B might be transported from the roots to the shoots, and the only elements that are actually reduced in the leaves are Mg, S, and Ca. Moreover, genotypic differences were associated with changes in the concentration of these elements under NaCl stress. Generally, L24 had much higher K levels in the roots, and higher Na, Ca, Cu, Mo, and Si levels in the leaves than X45. In the roots, the concentration of some elements (P, Ca, Cu, Fe, Mo, B, and Si) were lower in L24 than in X45, whereas in the leaves, these were higher in L24 than X45, particularly under high salt stress (SH). Moreover, the concentration of Mo and Si in the leaves increased with higher soil salinity in L24 but decreased in X45. Under salt stress, X45 accumulated mineral elements in the roots, whereas L24 transported mineral elements to the leaves. This indicated that the salt-tolerant variety had a better transport capacity of mineral elements than the salt-sensitive variety.

### Correlation analysis

**Fig 4:**
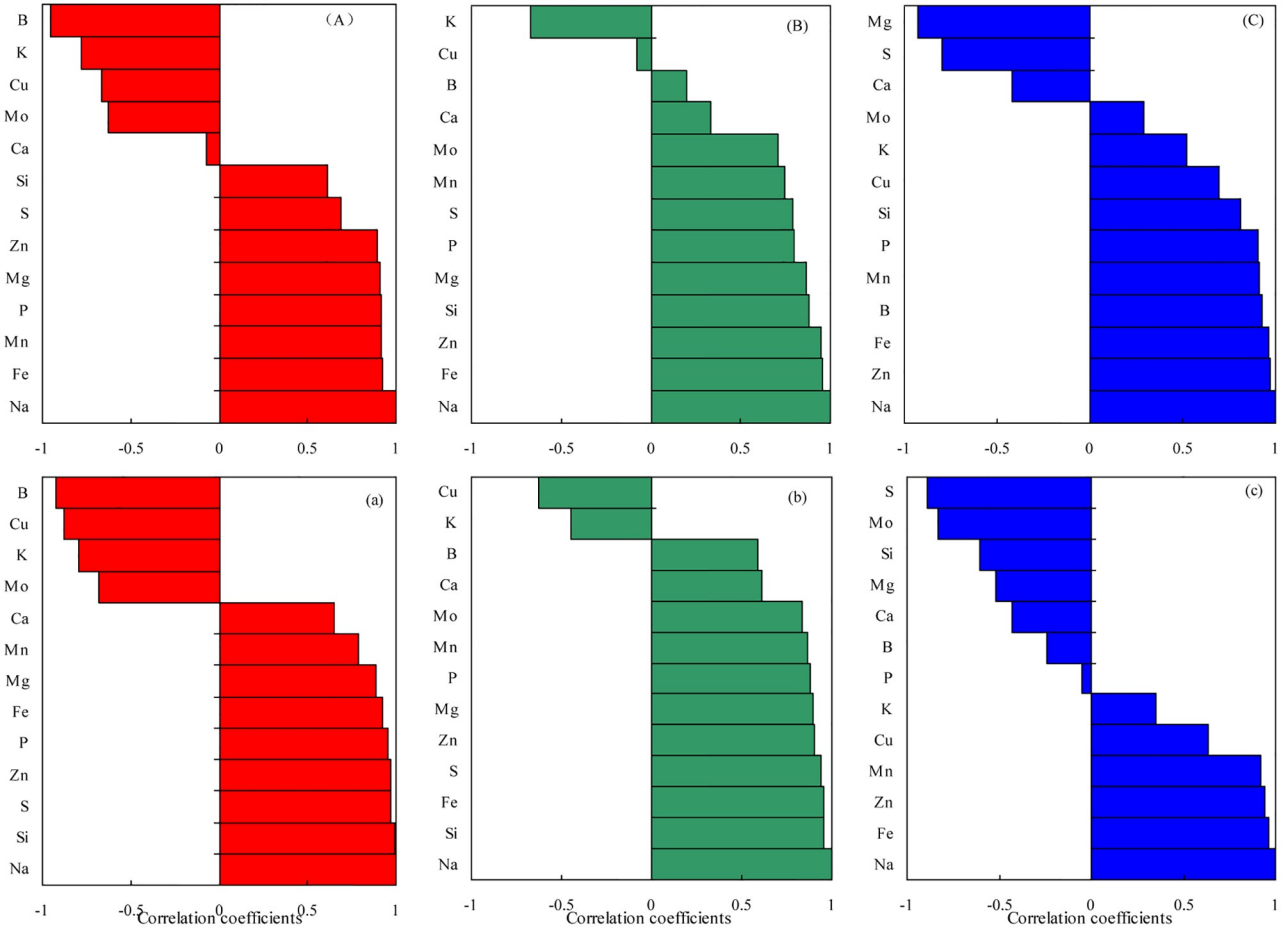
Correlation coefficients between Na and other elements under NaCl treatment. (A), (B), and (C) stand for the root, stem, and leaf of L24, respectively; (a), (b), and (c) stand for the root, stem, and leaf of X45, respectively.

Salt stress is mainly caused by excessive amounts of Na^+^ ions, and thus it is essential to understand the correlation between Na^+^ ions and other elements. Therefore, the correlation between Na and other elements in the roots, stems, and leaves under NaCl stress were analyzed (Fig 4). Na levels in the roots was negatively correlated with four elements (B, K, Cu and Mo) in both L24 and X45. In the stems, Na levels were significantly positively correlated with nearly all elements except for K in L24, and only Cu and K were negatively correlated with Na in X45. Na levels in the leaves were significantly negatively correlated with Mg, S, and Ca in L24, but had a significantly negative correlation with S, Mo, Si, Mg, Ca, and B in X45. The data show minimal difference between the roots and stems of the two varieties, whereas major differences were observed in the leaves. In the salt-sensitive cultivar, more elements were negatively related to Na than the salt-tolerant variety.

### Expression of the *GhSOS1*, *GhNHX1*, *GhHKT1*, and *GhAKT1* gene

**Fig 5:**
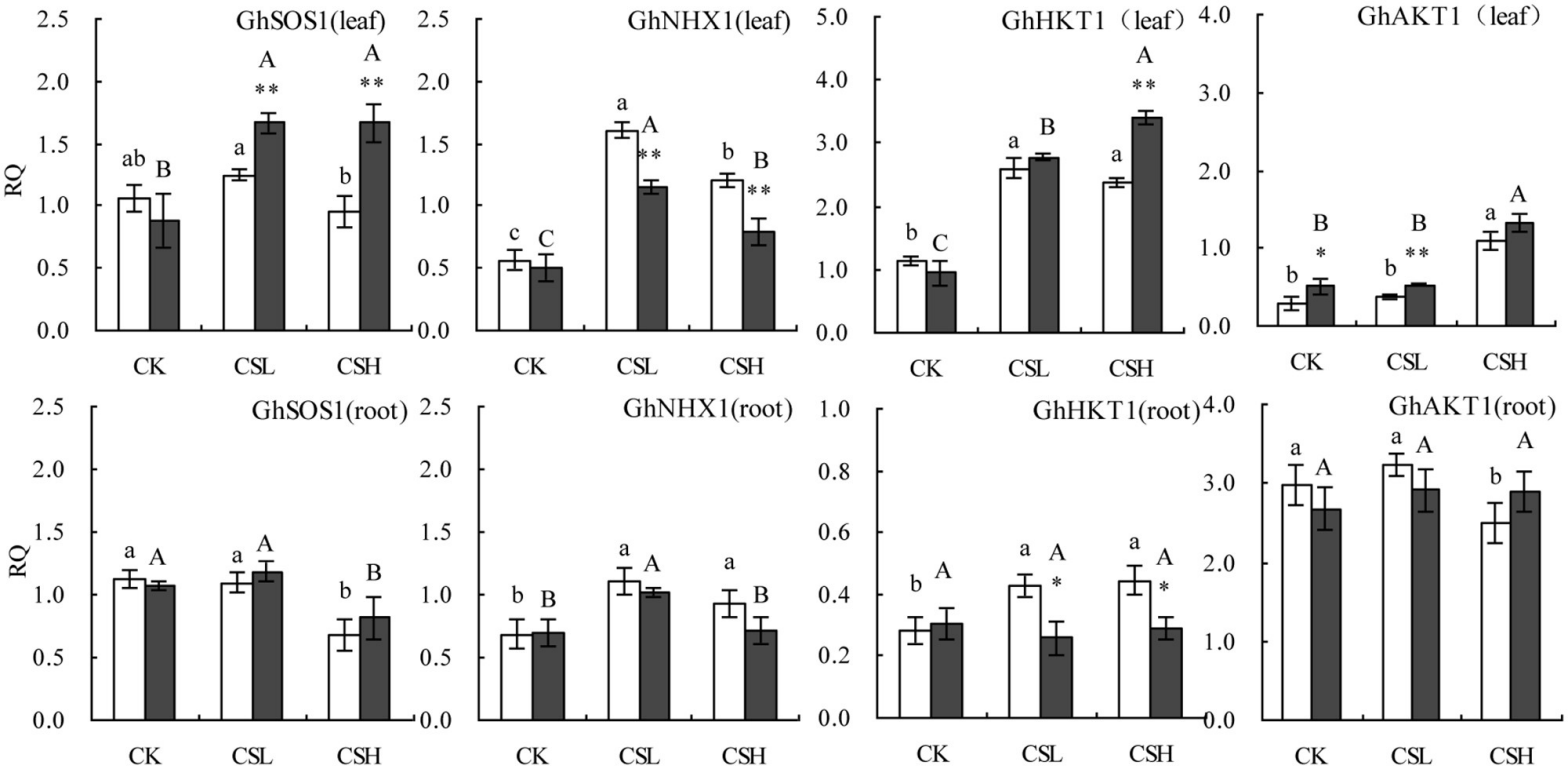
Relative expression of *GhSOS1*, *GhNHX1*, *GhHKT1*, and *GhAKT1* genes of cotton leaf and root under NaCl stresses. Symbols indicate L24 cultivar (white bar) and X45 cultivar (black bar). Vertical bars represent ± standard error (n = 3). Asterisks indicate a significant difference between L24 and X45 (*P<0.05; **P<0.01). Bars labeled with the different lowercase letters on open square bars or uppercase letters on closed square bars are significantly different (P<0.05).

To determine the changes in Na^+^ and K^+^ transport-related genes of the two varieties under salinity stress, the expression patterns of *GhSOS1*, *GhNHX1*, *GhHKT1*, and *GhAKT1* were analyzed by qRT-PCR (Fig 5). The expression of *GhSOS1* in X45 gradually increased during salt exposure but decreased in the leaves of L24. In the roots, the expression levels of *GhSOS1* in the two varieties decreased with increasing stress levels. However, there are no difference between CK and CSL. *GhNHX1* initially increased and then decreased with increasing salt stress in the roots and leaves in the two varieties However, there are no significant difference between CSL and CSH in roots of L24. Compared with L24 and X45, the expression level of *GhSOS1* and *GhNHX1* in the roots did not significantly differ between the two varieties, but in the leaves, *GhSOS1*’s expression level in X45 was higher than L24. By contrast, *GhNHX1*’s expression level in L24 was higher than that of X45. These data suggest that the two cultivars response to salt stress differently; Na^+^ efflux capacity of X45 is higher than that of L24, whereas Na^+^ vacuolar sequestration ability of L24 is higher than that of X45. The two K potassium transporters, *GhHKT1* and *GhAKT1*, are considered as the major routes for K^+^ uptake; the expression levels of these two genes in the leaves increased with higher salt stress. In addition, their expression levels in X45 were all higher than L24. However, in the roots, a complete opposite profile was observed, wherein the expression level of *GhHKT1* in L24 was significantly higher than X45. The change in *GhAKT1* expression in the roots did not largely differ between the two cultivars. This explains why the salt-tolerant cultivar L24 had a higher leaf Na^+^ content and accumulated more K^+^ in the roots.

### Transcriptomic analysis

To obtain a general overview of the cotton transcriptome and an initial comparison between Salt-tesistant (L24) and salt-susceptibel (X45) cotton transcripts, six librires (X45.CK, L24.CK, X45.SL, L24.SL, X45.SH, L24.SH) were constructed for paied end sequencing (Illumina sequencing). After filtration of low-quality and adapter sequences, an average of 6.02 Gb of Clean Data was obtained for each sample, Q30 base percentage is above 85.00%. These processed high-quality paired end readings will be used for further analysis (S2 Table).

Gene expression levels were estimated by fragments per kilo base of transcript per million fragments mapped (FPKM). Differential expression analysis of the treatment and control groups was performed using DEGseq. A cutoff *P* value < 0.01 adjusted by false discovery rate (FDR) and a fold change≥2 were used in identifying differentially expressed genes (DEGs). A total of 1,178 and 1,101 DEGs were obtained in different cultivars’ leaves under SL and SH treatments, respectively. The number of DEGs in response to salt stress in the two cotton genotypes was significantly different (Table 2). There were 807 and 303 DEGs in X45 under low concentration salt stress, and 382 and 197 DEGs in L24 that showed significant upregulation and downregulation under low-concentration salt stress compared with the controls, respectively. In addition, 40 and 172 DEGs in X45 and 507 and 409 DEGs in L24 showed significant upregulation and downregulation under high-concentration salt stress, respectively. Under the low NaCl stress, the expression of differentially expressed genes in X45 was 2.11-fold higher than those in L24. However, under the high NaCl stress, differentially expressed genes in L24 were 4.32-fold greater than those in X45. In L24, the number of upregulated genes was greater than the number of downregulated genes, whereas in X45, the number of downregulated genes exceeded the number of upregulated genes. These results indicate that the salt-sensitive cultivar suffered from salt stress earlier than salt-tolerant cultivar.

**Table 2:** The number of differentially expressed genes in leaves.

Combination	ALL DEG	Up regulated	Down regulated
X45 SL VS CK	807	303	504
L24.SL VS CK	382	197	185
X45.SH VS CK	212	40	172
L24.SH VS CK	916	507	409

Unigenes with false discovery rate (FDR) no greater than 0.01 and fragments per kb of transcript per million fragments mapped (FPKM) between samples with fold-change (FD) ≥ 2) were considered differentially expressed genes (DEGs).

**Fig 6:**
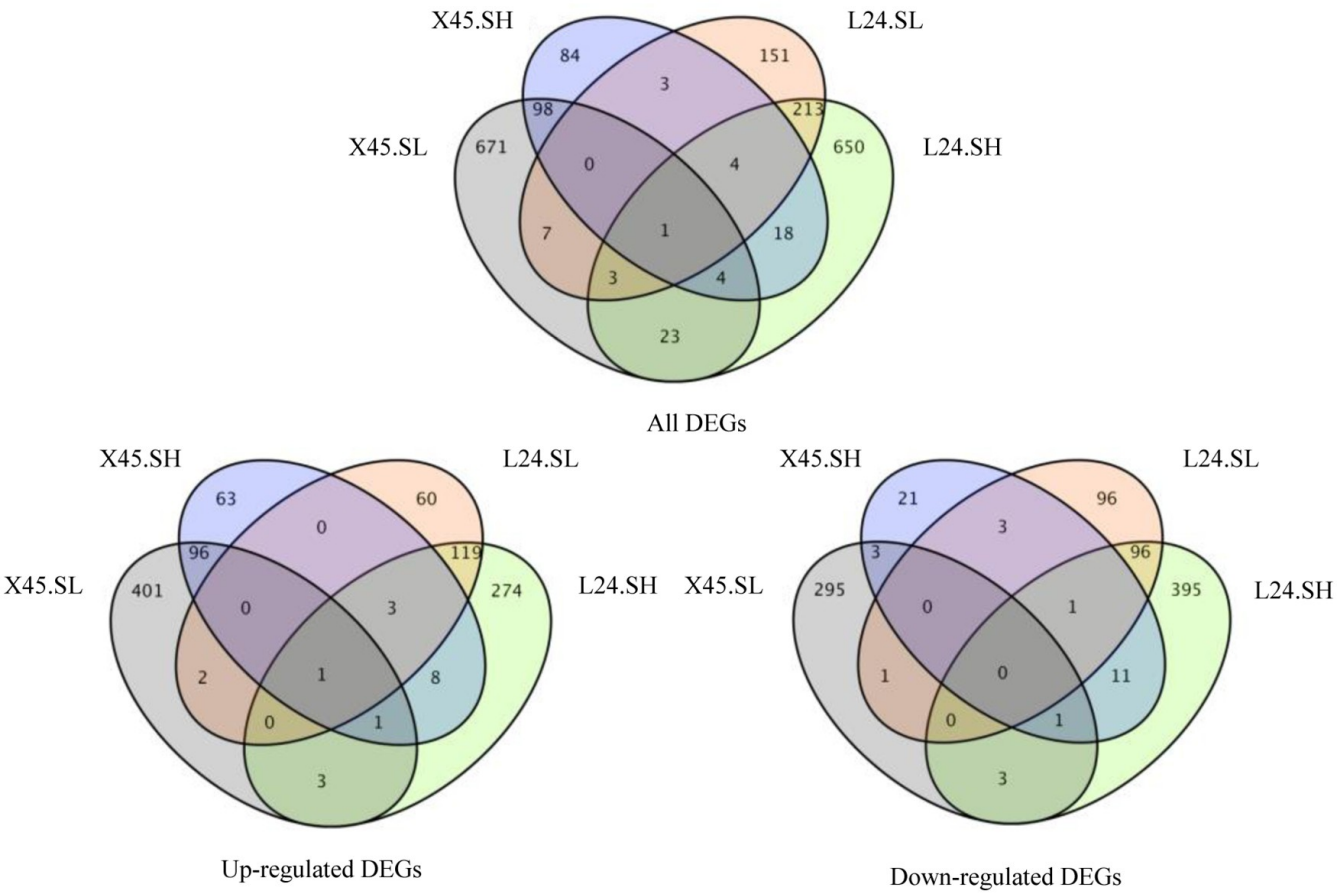
Venn diagram of the number of differentially expressed genes (DEGs) (log2>1.5) treated with salt stress in different cultivars compared with the controls.

The DEG sets of the SL and SH treatments using the two cultivars were analyzed separately (Fig 6). Under low-concentration NaCl stress, 11 differentially co-expressed genes were identified in both cultivars, and only 1 upregulated gene and 3 down-regulated genes. Under the high-concentration NaCl treatment, 27 differentially co-expressed genes were identified (13 up regulated; 13 downregulated). These results indicate that there are significant differences in salt tolerance between the two varieties, and that cotton has different salt tolerance strategies in response to low- and high-slat stress.

Using COG functional classification, 170 and 402 DEGs in L24 after SL and SH salt stress were predicted respectively (S1 Fig). We also found some functional annotation information associated with ion homeostasis that are involved in inorganic ion transport and metabolism (9 genes distributed in SL, and 21 genes in SH), signal transduction mechanisms (11 and 42), and energy production and conversion (15 and 29). Furthermore, COG functional annotation revealed that in X45, 337 DEGs were predicted in SL, and 78 in SH. Functional annotation information associated with ion homeostasis such as inorganic ion transport and metabolism (19 genes distributed in SL, and 2 genes in SH), Signal transduction mechanisms (58 and 25), and energy production and conversion (13 and 2). GO annotation revealed that for all DEGs, three major categories were highly enriched after salt treatment, which included biological process, molecular function, and cellular component (S2 Fig). 691 (85.63%) and 280 (73.30%) DEGs in X45 and L24 under SL were annotated, respectively. 179 (84.43%) and 711 (77.62%) DEGs in X45 and L24 under SH were annotated, respectively (S3 Table). GO annotation revealed that the DEGs were related to the maintenance of ion balance (S4 Table), such as ion transport and homeostasis, ATPase coupled to transport, vacuolar function, and element-related regulation (sodium, potassium, calcium, magnesium, manganese, cadmium, iron, zinc, copper, phosphate, silicate, sulfate and sulfur, selenate and selenium, boron and borate).

**Fig 7:**
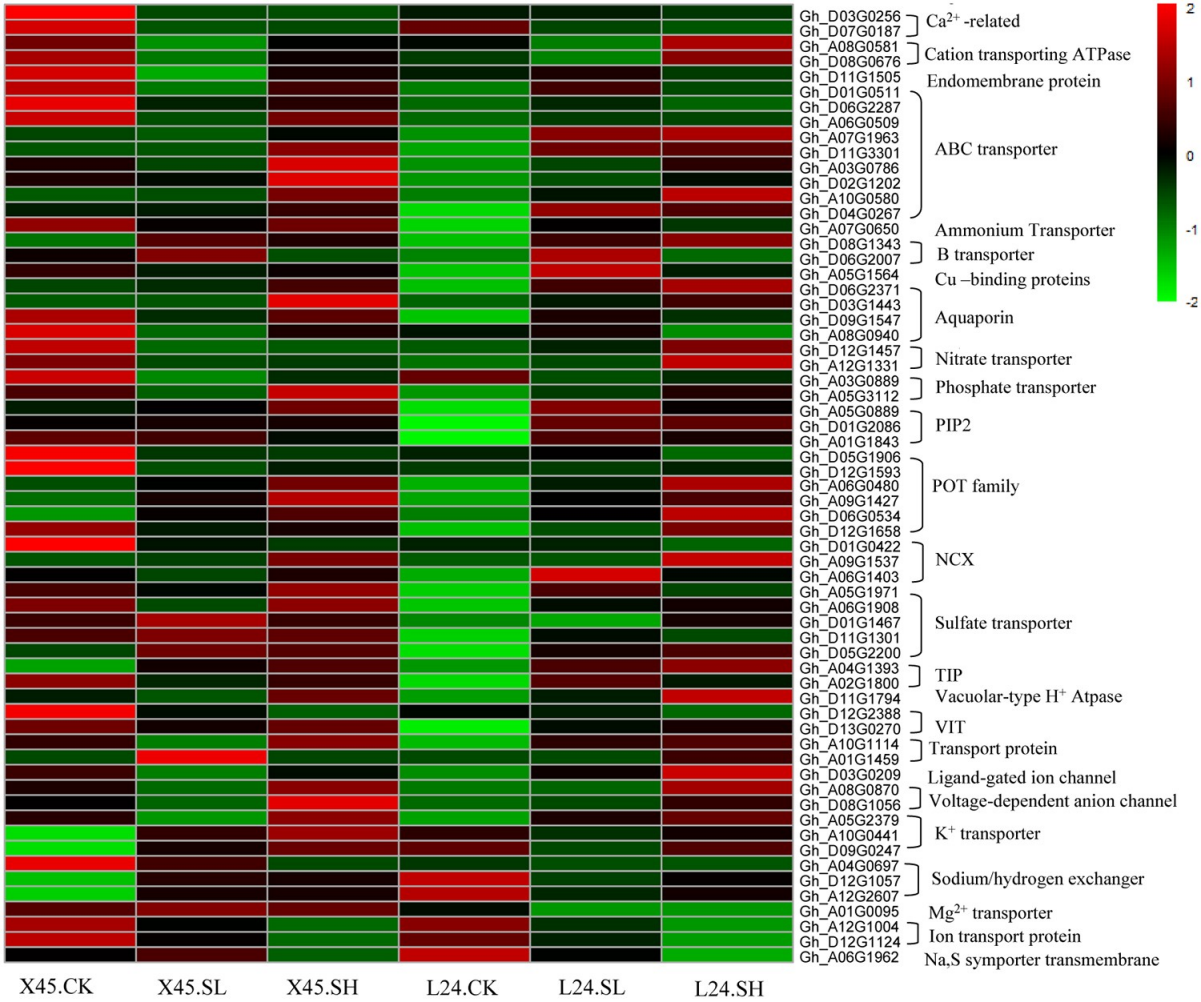
Heat map for the DEGs related to transporter proteins in the leaves of two cotton cultivars under NaCl stress. The abscissa represents the sample name, and the ordinate represents the differentially expressed gene. Different columns in the figure represent various samples, and rows represent genes. The color represents the level of expression of the gene (log_2_ (FPKM+1) in the sample. Gene annotations shown in the heat map are obtained from the cotton gene database (https://www.cottongen.org/).

DEGs related to ion transport and homeostasis in the two cultivars in response to salt stress were identified (Fig 7). These included genes encoding Ca^2+^ transport-related proteins D03G0256 and D07G0187, an intracellular endomembrane protein that functions as a channel or small molecule transporter (D11G1505), a Mg^2+^ transporter (A01G0095), a transmembrane sodium:sulfate symporter (A06G1962), and two ion transporter proteins (A12G1104, D12G1124). The genes were significantly downregulated in both X45 and L24. Most genes related to ion transport were upregulated in L24, but not in X45, such as the ones encoding ABC transporter (A07G1963, A06G0509, d01G0511, D04G0267), ammonium transporter family (A07G0650), boron transporter (D08G1343), copper-binding proteins (A05G1564), aquaporin (D06G2371, D03G1443, D09G1547), and PIP2 protein (D01G2086, A01G1843) that plays a very important role in signal transduction and membrane protein function regulation, POT family (proton-dependent oligopeptide transporter) (A06G0480, A09G1427, D06G0534, D12G1658), NCX (Sodium/calcium exchanger protein) (A09G1537, A06G1403), sulfate transporter (A05G1971, A06G1908, D01G1467, D11G1301, D05G2200), tonoplast intrinsic protein (TIP) (A04G1393, A02G1800), VACUOLAR-TYPE H^+^ ATPASE (D11G1794), vacuolar iron transporter (VIT) 1-like protein)(D13G0270), transport protein (A10G1114, A01G1459), ligand-gated ion channel (D03G0209), and voltage-dependent anion channel (A08G0870, D08G1056). In particular, two genes encoding nitrate transporter (D12G1457, A12G1331), and cation-transporting ATPase (A08G0581, D08G0676) exhibited the opposite expression trend in the two varieties, i.e., significantly upregulated expression in L24 and dramatically downregulated expression in X45. By contrast, genes encoding K^+^ potassium transporter (A10G0441, D09G0247) and sodium/hydrogen exchanger (D12G1057, A12G2607) were significantly upregulated in X45, but not in L24. One sodium/hydrogen exchanger family gene (A04G0697) was significantly downregulated in L24.

### qRT-PCR validation

**Fig 8:**
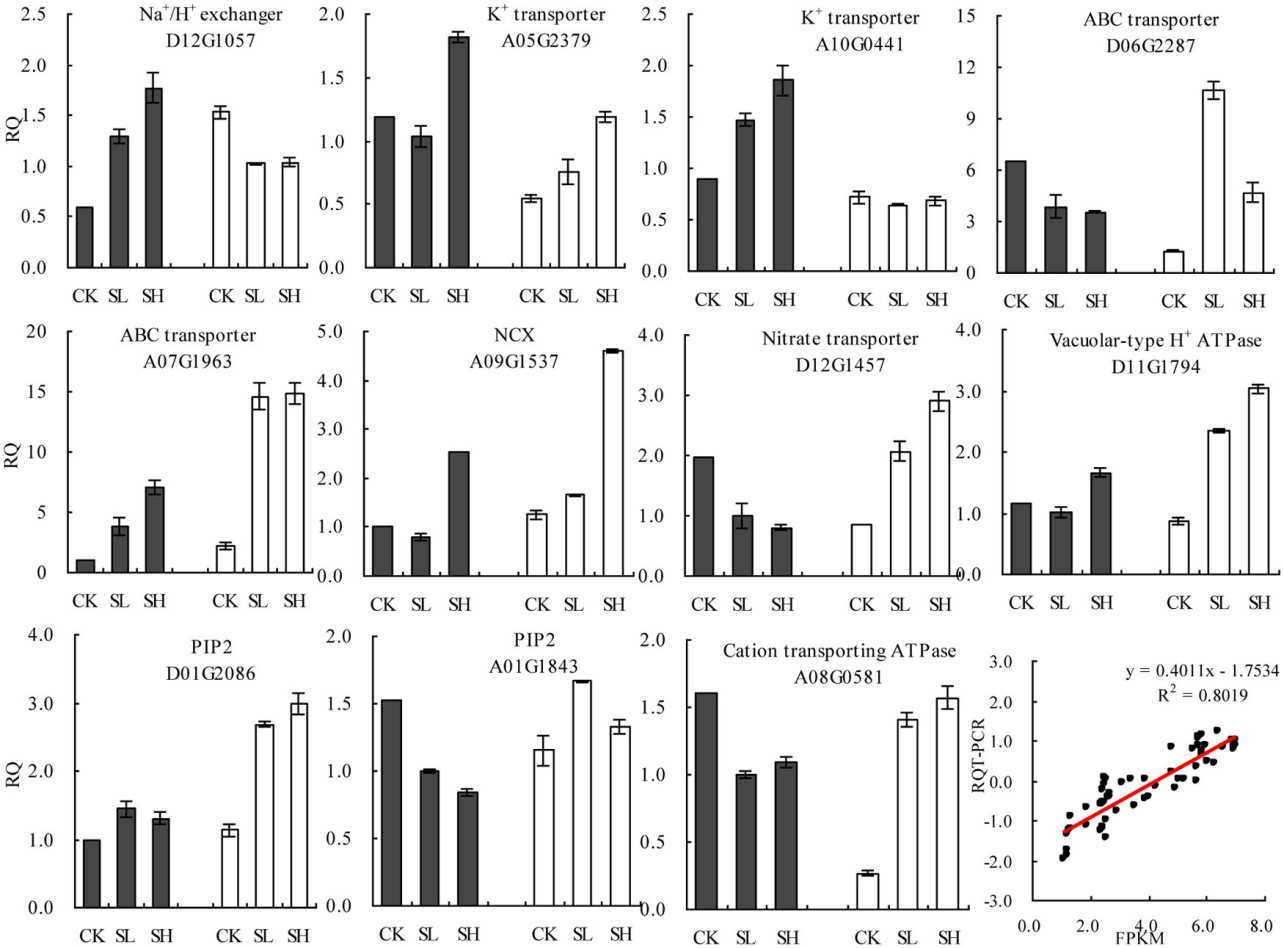
Validation of RNA-Seq results by qRT-PCR analysis. qRT-PCR expression data of 11 genes of X45 cultivar (black bar) and L24 cultivar (white bar). Error bars represent the SD of three biological replicates and Comparison between the log_2_ of gene expression ratios obtained from RNA-seq data and q RT-PCR.

To validate our sequencing data, 11 DEGs in both genotypes were subjected to qRT-PCR analysis (Fig 8). The qRT-PCR findings coincided with the transcriptome (Illumina) sequencing results, and their correlation coefficient was R^2^ = 0.8091, indicating that the transcriptome sequencing results were reliable.

## Discussion

As one of the major environmental stress factors, salinity negatively affects the growth and yield of crop plants. Growth inhibition is plants’ the most typical physiological response to saline habitats [29, 30]. Several groups have reported that the biomass of cotton plants might be affected by saline conditions [31, 32]. The results of this study showed that NaCl stress significantly impaired total biomass (leaves, roots, and stems) in the two tested cultivars (salt-tolerant genotype L24 and salt-sensitive genotype X45), as the levels were significantly lower than the control. The observed reduction in total biomass index exceeded 48% with increasing salt levels from 0.17 dS·m^−1^ to 1.39 dS·m^−1^, and differences among cotton cultivars were significant. Similarly, Akhtar *et al*. [30], Basal [32], and Munis *et al*. [33] found that biomass production was negatively affected by NaCl application in cotton. The observed reduction in biomass resulting from the increasing in soil salinity might be attributable to the combinatorial effect of osmosis and Cl^−^ and Na^+^ ions [32, 34]. Our study also showed that NaCl stress mainly inhibited shoot growth at low concentrations, whereas high concentrations significantly inhibited the growth of shoots and roots. These findings also indicated that shoot growth is generally more sensitive than root growth in response to salinity [35]. The growth inhibition rate of X45 under NaCl stress was significantly higher than L24. The inhibitory effect of salt on crop growth might be due to the toxicity of sodium ions [8, 36, 37]. Salt stress is known to inhibite the growth of cotton, and the relative biomass of plants was considered a reliable index, as it reflects growth performance under salt stress [38].

Salt stress reduces the metabolism of mineral during plant growth as well as inhibits the absorption of nutrients by plants [31, 39, 40]. In addition, salt stress influences nutrient availability, competitive uptake, transport, or partitioning in plants, likely resulting in nutrient imbalances [41]. Homeostasis can be defined as the tendency of an organism to maintain an internal steady state, and thus rebuilding intracellular ion homeostasis under salt stress is an important salt tolerance strategy for plants [42]. Wu *et al*. [10] used ICP-AES to analyze the composition of 10 elements in three barley genotypes and found that the ionome in the roots and shoots of barley were rearranged under moderate and high salt stresses conditions. Under salt stress, plants frequently accumulate large amounts of Na to lower the cell water potential [7, 43]. In agreement with previous studies, we found that cotton plants also accumulated large amounts of Na under salt stress. In addition, the Na concentration in the leaves was higher than the roots under high salt stress, indicating that neither salt-tolerant nor salt-sensitive cotton could prevent Na transport from the roots to leaves. Moreover, more Na accumulated in the L24 leaves than the X45 leaves, indicating that cotton plants tolerated salt stress by storing Na in the leaves. These findings coincided with the upregulation of *GhNHX1* in L24 leaves. Plants avoid excessive Na^+^ accumulation by increasing Na^+^ efflux and/or increasing vacuolar sequestration of Na^+^ [44]. *Salt overly sensitive-1* (*SOS1*) and *NHX* are two key salt tolerance genes, and *SOS1* mainly regulates the plasma membrane (PM) Na^+^/ H^+^antiporter, which can exclude excess Na^+^ from cytoplasm [45]. *NHX1* is a vacuolar membrane-bound Na^+^/H^+^ antiporter, which can transport Na^+^ from the cytoplasm into vacuoles [6]. Previous studies have shown that the overexpression of *NHX1* [46, 47] or *SOS1* [48, 49] in transgenic plants can increase salt tolerance. Our result found that L24 and X45 have different Na^+^ regulatory strategies. Na^+^ efflux capacity of X45 is higher than that of L24, whereas Na^+^ vacuolar sequestration ability of L24 is higher than that of X45. In this study, K levels in the roots decreased with increasing NaCl concentration; however, NaCl stress increased the K concentration in leaves. qRT-PCR and transcriptomics analysis also found that the K potassium transporter (*GhHKT1*, *GhAKT1*, A10G0441, D09G0247 in X45; A05G2379 in L24) was significantly upregulated in the leaves under salt stress. Low affinity K^+^ transporter (*AKT1*) and high affinity K^+^ transporter are considered the major routes for K^+^ uptake and play critical roles in maintaining K^+^/Na^+^ balance in salt-stressed plants [50, 51]. The moderate accumulation of Na in the leaves may also cause a slight increase in K levels [52]. A high K/Na ratio has been considered as a salt tolerance mechanism of crops [53]. Although there was no significant difference in the Na concentration between the roots of L24 and X45, the K/Na ratio of L24 roots was higher than that of the X45 roots.

Salt inhibits the uptake of other ions, leading to decreased P, K, Ca, and Mg concentrations in the leaves and roots [54–59]. However, several reports have shown that K, Ca, and S are stable in the leaves, leading to lower K/Na ratios [59–61]. Thomas [60] reported that salt increased Ca and Mg levels in leaves. However, Rathrtt [62] reported that the increase in Na levels in plant cells results in a moderate decrease in Ca and Mg levels. Our study found that NaCl stress significantly increased P levels in the roots, and the P concentration in the leaves also increased to a certain extent compared with CK treatment. For example, P levels in the L24 leaves increased under high salt stress, and transcriptomics analysis also found that the phosphate transporter encoding gene (A05G3112) was significantly upregulated in L24 leaves under high salt stress. Maintaining a certain concentration of Ca in plant leaves is of great importance for salt tolerance in cotton. Ca and Na have certain antagonistic effects, and excessive Na intake leads to a relative deficiency of Ca in cotton plants [63]. Zhang et al. [64] found that salt stress significantly decreased Ca levels in plants. Hirayama [65] also found that high Na levels could interfere the binding Ca in plasma membrane and cell membrane system, eventually destroying the integrity and function of the membrane structure. Our study also showed that NaCl stress (high concentrations) decreased the Ca levels in the leaves. In addition, NaCl stresses decreased the Mg and S levels in the leaves. Transcriptomics analysis also found that Ca transport-related genes (D03G0256, D07G0187) were significantly downregulated in both cultivars, whereas Mg transporter protein (A01G0095) and sodium:sulfate symporter transmembrane (A06G1962) were significantly downregulated in L24 and X45 under high salt stress. The significant decrease in Mg levels in the leaves might be due to the lower chlorophyll content in the leaves after salt stress [64]. The absorption of Ca, Mg, and S in different cotton genotypes vary, and in general, the Ca, Mg, and S levels in cotton organs were as follows: leaves > stems > roots. In addition to K, Ca and Mg are also important for improving salt tolerance of cotton [13, 66]. However, Severino *et al*. [14] showed that Ca and Mg could not reduce Na^+^ toxicity at the seedling stage of cotton. According to a previous report, Mn, Fe, Zn, and Cu levels in wheat and maize significantly decreased under NaCl stress [67, 68]. Our study found that NaCl stress significantly reduced the Cu, B, and Mo levels in the roots, but increased the Zn, Mn, Fe, and Si levels in the roots and Zn, Mn, Fe, Cu, and B levels in the leaves. The Zn, Mn, Fe, Cu, B, Mo, and Si levels in the leaves of L24 increased under high salt stress. Transcriptomics analysis also showed that cation transporting ATPase encoding genes (A08G0581, D08G0676) and boron transporter encoding genes (D08G1343, D06G2007) were upregulated in L24 under high salt stress. The reason for the increase in Fe levels under salt stress may be that in order to maintain growth, cotton synthesizes chlorophyll to improve photosynthesis to respond to high salt stress. Mn plays a catalytic role in chlorophyll synthesis, which is closely related to photosynthesis and respiration of plants. This study found that Mn levels in cotton leaves significantly increased under salt stress, but Karimi *et al*. [69] reported that excessive accumulation of Na reduced the absorption of Mn. Si is a beneficial element, and salt stress also significantly affected the absorption of Si in cotton. This study showed that NaCl stress significantly increased the Si levels in X45 roots and L24 leaves. Li *et al*. [70] studied the effects of Si on tomato seedling growth under salt stress and found that Si significantly alleviated the adverse effects of salt stress on tomato seedling growth, photosynthetic performance, and soluble protein levels. The PCA results showed that the greatest contributory elements in the first principal component were Na, Mg, S, P, and Si in the roots and Na, Mg, Zn, Fe, and Mn in the leaves. In addition, NaCl stress increased the levels of Na, Mg, P, S, and Si in the roots and increased Na, Zn, Fe, and Mn levels in the leaves; however, NaCl stress decreased the Mg levels in the leaves. From the second principal component, the greatest contributory elements were Ca, Cu, Zn, and Mo in the roots and Ca and Mo in the leaves. Generally, NaCl stress increased the Zn levels in the roots of L24 and X45 and the Cu levels in the leaves of L45. Moreover, low-concentration NaCl stress increased the Ca levels in the leaves of L24 and X45. However, NaCl stress decreased the Cu and Mo levels in the roots of L24 and X45 and decreased the Mo levels in the leaves of X45. Because the level of salt tolerance is relative, the observed inconsistencies might be related to the use of different cotton cultivars, genotypes, growth stages, or evaluation methods. In our study, NaCl reduces the concentrations of certain minerals and increases that of others, and the patterns depend on the minerals, the plant part, and varieties being compared to the control. Transcriptome analysis showed that different genotypes of cotton adapt to salt stress by upregulating and downregulating the expression of genes to re-establish ion homeostasis. Therefore, our results may be helpful in elucidating the molecular mechanisms of salt stress response in different cotton genotypes.

## Conclusions

Overall, NaCl stress reduced cotton biomass and low NaCl stress inhibited shoot growth, whereas high NaCl stress inhibited both root and shoot growth. NaCl stress inhibits the growth of the salt-sensitive cultivar (X45) more effectively than that of the salt-tolerant cultivar (L24). NaCl stress promoted the Na levels in the roots, stems, and leaves, and reduced the Mg, S, and Ca levels in the leaves. K, Cu, and B are transported from the roots to the shoots to resist salt stress. The levels of certain elements (P, Ca, Cu, Fe, Mo, B, and Si) were lower in the roots of L24 than X45, but levels in the leaves were the opposite (higher levels in L24 than X45). The salt-sensitive cultivar (X45) had more elements that were negatively related to Na than the salt-tolerant cultivar (L24). Most genes related to ion transport and homeostasis were upregulated in L24, but not in X45. The two varieties respond to salt stress differently; Na^+^ efflux capacity of X45 is higher than that of L24, whereas Na^+^ vacuolar sequestration ability of L24 is higher than that of X45. Cotton adapts to NaCl stress by re-establishing ionic homeostasis by transporting mineral elements to the leaves.

## Supporting information

S1 Fig COG classification of the differentially expressed genes in the leaves of L24 and X45 after low and high salt treatments. (DOCX)

S2 Fig GO classification of DEGs between two cotton cultivars under low salt stress and high salt stress. (DOCX)

S1 Table Primers used for qRT-PCR analysis. Primers name, annotion, abbreviation used for analysis of genes expression.
(XLS)

S2 Table Characteristics of clean reads in six libraries. (DOCX)

S3 Table The number of DEGs of X45 and L24 under SL/CK and SH/CK. (XLS)

S4 Table The number of DEGs related to ion homeostasis of X45 and L24 under SL/CK and SH/CK. (XLS)
