# Supplementary material for: Ionomic and transcriptomic analyses of two cotton cultivars (Gossypium hirsutum L.) provide insights into the ion balance mechanism of cotton under salt stress
Source: PLoS One. 2019 Dec 23;14(12):e0226776. doi: 10.1371/journal.pone.0226776 (PMC6927655; doi:10.1371/journal.pone.0226776)
Supplement: S1 Fig — (DOCX) [file pone.0226776.s001.docx]

**Figure S1. COG classification of the differentially expressed genes in the leaves of L24 and X45 after low and high salt treatments.**
