# Supplementary material for: Ionomic and transcriptomic analyses of two cotton cultivars (Gossypium hirsutum L.) provide insights into the ion balance mechanism of cotton under salt stress
Source: PLoS One. 2019 Dec 23;14(12):e0226776. doi: 10.1371/journal.pone.0226776 (PMC6927655; doi:10.1371/journal.pone.0226776)
Supplement: S2 Fig — (DOCX) [file pone.0226776.s002.docx]

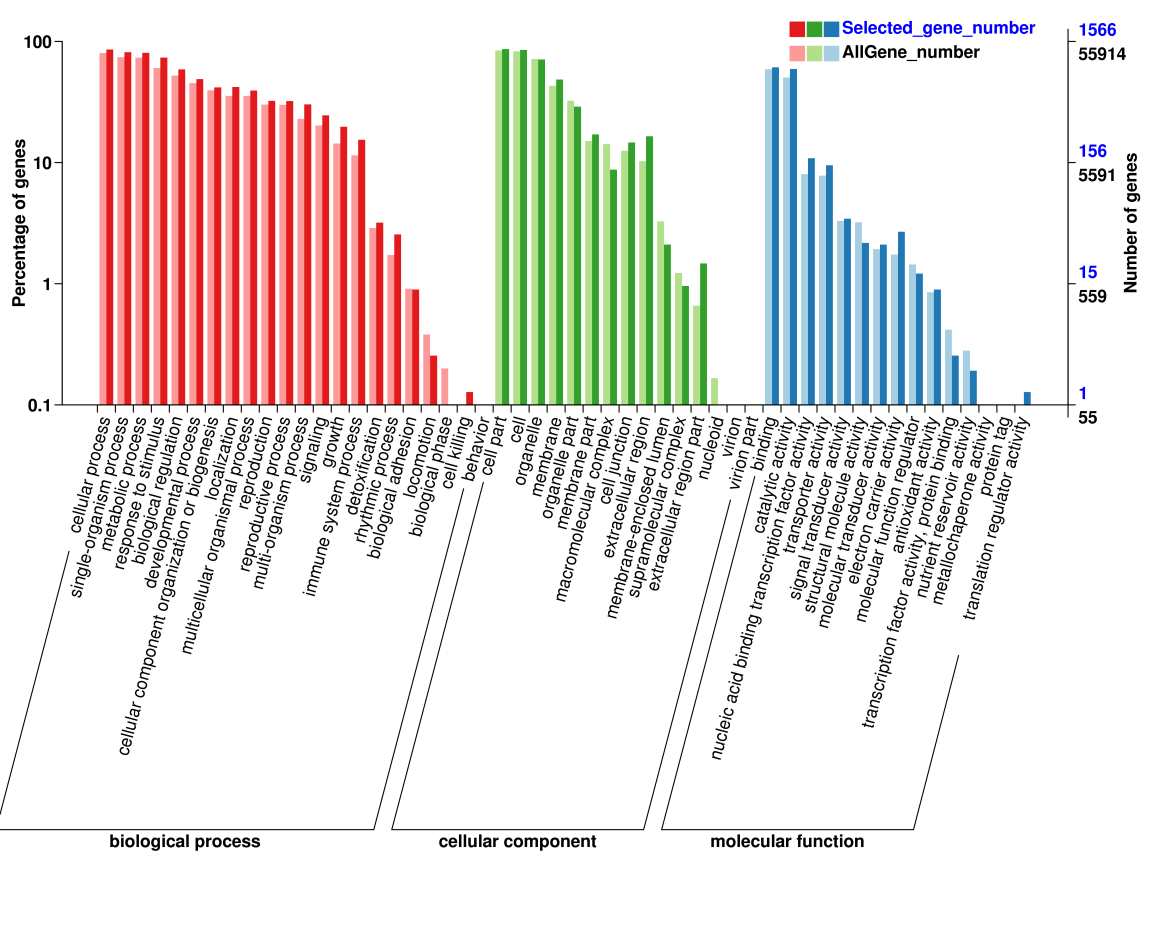


**Figure S2. GO classification of DEGs between two cotton cultivars under low salt stress and high salt stress.** X-axis represents the GO terms names: red pillars represent of biological process, green pillars represent cellar component, and blue pillars represent molecular function. The deeper colours represent DE genes, the lighter colours represent all genes. The left Y-axis represents percentage of genes, right Y-axis represents number of gene.
