# Supplementary material for: Ionomic and transcriptomic analyses of two cotton cultivars (Gossypium hirsutum L.) provide insights into the ion balance mechanism of cotton under salt stress
Source: PLoS One. 2019 Dec 23;14(12):e0226776. doi: 10.1371/journal.pone.0226776 (PMC6927655; doi:10.1371/journal.pone.0226776)
Supplement: S2 Table — (DOCX) [file pone.0226776.s004.docx]

Table S2 Characteristics of clean reads in six libraries

| Treatment | [variety](javascript:;) | Clean Reads | GC Content | %≥Q30 | Mapped Reads |
| --- | --- | --- | --- | --- | --- |
| CK | X45 | 23,836,720 | 44.80% | 85.05% | 76.58% |
|  | L24 | 23,277,378 | 44.45% | 90.00% | 83.11% |
| SL | X45 | 21,316,718 | 44.10% | 86.05% | 79.41% |
|  | L24 | 27,465,223 | 44.54% | 89.86% | 82.56% |
| SH | X45 | 23,261,166 | 44.06% | 86.26% | 79.33% |
|  | L24 | 22,113,938 | 44.28% | 85.32% | 77.01% |

Clean reads: Total number of pair-end Reads in Clean Data; GC content: G and C bases as a percentage of total bases in Clean Data; ≥Q30%: Clean Data with a base value of 30 or greater; Mapped Reads: Number of Reads aligned to the reference genome as a percentage of Clean Reads;
